# Longitudinal Study of Viral and Bacterial Contamination of Hospital Pediatricians’ Mobile Phones

**DOI:** 10.3390/microorganisms8122011

**Published:** 2020-12-16

**Authors:** Aymeric Cantais, Florence Grattard, Julie Gagnaire, Olivier Mory, Aurélie Plat, Manon Lleres-Vadeboin, Philippe Berthelot, Thomas Bourlet, Elisabeth Botelho-Nevers, Bruno Pozzetto, Sylvie Pillet

**Affiliations:** 1 Paediatric Emergency Department, University Hospital of Saint-Étienne, 42000 Saint-Étienne, France; aymeric.cantais@chu-st-etienne.fr (A.C.); olivier.mory@chu-st-etienne.fr (O.M.); aurelie.cantais@chu-st-etienne.fr (A.P.); 2 GIMAP (Groupe Immunité des Muqueuses et Agents Pathogènes) EA-3064, Medicine Faculty of Saint-Etienne, Campus Santé-Innovations of Saint-Etienne, 42270 Saint-Priest-en-Jarez, France; florence.grattard@chu-st-etienne.fr (F.G.); philippe.berthelot@chu-st-etienne.fr (P.B.); thomas.bourlet@chu-st-etienne.fr (T.B.); elisabeth.botelho-nevers@chu-st-etienne.fr (E.B.-N.); bruno.pozzetto@chu-st-etienne.fr (B.P.); 3 Laboratory of Infectious agents and Hygiene, University Hospital of Saint-Étienne, 42000 Saint-Étienne, France; manon.lleres-vadeboin@chu-st-etienne.fr; 4 Hygiene Department, University Hospital of Saint-Étienne, 42000 Saint-Étienne, France; julie.gagnaire@chu-st-etienne.fr; 5 Infectious Diseases Department, University Hospital of Saint-Étienne, 42000 Saint-Étienne, France

**Keywords:** cell phones, infection control, cross infection, infectious disease outbreak

## Abstract

Mobile phones (MPs) of healthcare workers (HCWs) may represent an important source of transmission of infectious agents. This longitudinal study documents the contamination of these tools. Ten MPs handled by senior pediatricians were sampled once a week during 23 weeks in three pediatric wards of the University Hospital of Saint-Etienne, France. Cultures were performed for bacteria and multiplex PCR assays for a panel of respiratory and enteric viruses. A questionnaire on hygiene habits regarding phoning and care was filled-in by pediatricians before and after the study. From a total of 230 samples, 145 (63%) were contaminated by at least one pathogen. The MPs from emergency departments were the most impacted. Viruses were detected in 179 samples; bacteria were isolated in 59 samples. Contamination increased during the winter epidemic peak. A cross-contamination by *Paracoccus yeei* between hands and MPs of different HCWs was demonstrated. The communication of the study results influenced the hygiene behaviors. This study highlights the contamination of MPs by pathogens that are resistant in the environment, and its sustainability along the winter season. The role of MPs as vectors of nosocomial infection needs to be better investigated.

## 1. Introduction

Mobile phones (MPs), routinely used by healthcare workers (HCWs), improve the quality and efficiency of communications in healthcare settings [1], including high-risk departments [2,3]. Often carried by HCWs, they can contribute to nosocomial pathogens transfer via hands [4]. Contamination of MPs used by HCWs has been reported with bacteria, including multiple antibiotic-resistant strains [4,5,6,7,8,9]. Although the risk of MPs as vectors of nosocomial infection exists [5,7], they are rarely cleaned [10,11,12].

The contamination of MPs by viruses has been poorly investigated [9]. The presence of rotavirus RNA on MPs of paediatricians facing an outbreak with this agent has been reported during a one-day study performed in our centre [11]. The persistence of viruses on surfaces [13,14], on hands [15,16] as well as the transfer from fomites to hands (and vice-versa), has been demonstrated [16,17,18], especially in paediatric wards [12], and has been implicated in the spread of enteric [19] and respiratory viruses [20].

To the best of our knowledge, studies looking at the co-detection of a large panel of viruses and bacteria on the MPs of HCWs are missing [9]. In this prospective longitudinal study, we aimed to evaluate the contamination by infectious agents of MPs handled by paediatric senior physicians during a 23 week period including the 2015–2016 winter season, together with its impact on the hygiene habits of the paediatricians on care.

## 2. Materials and Methods

### 2.1. Design of the Study

This prospective longitudinal study was performed at the University Hospital of Saint-Etienne, France. Ten professional MPs, all used by senior paediatricians only and at work only, were selected and sampled weekly for 23 weeks, from December 2015 to May 2016, to analyse the bacterial and viral contamination recovered from their surface. This period including the winter season was chosen because of the circulation of epidemic respiratory and enteric viruses in our setting [21,22]. Three departments were targeted: the paediatric intensive care unit (PICU), the general paediatric hospitalization unit (GPHU) and the paediatric emergency department (PEMD); the paediatricians were all volunteers.

All MPs were similar digital enhanced cordless telecommunication (DECT) phones with keypads (Funkwerk^®^ FC4), used daily and only on site. From the 10 MPs, 8 were professional nominative devices from paediatricians working in the PICU (*n* = 3), in the GPHU (*n* = 3) or in the PEMD (*n* = 2). The last 2 professional MPs were shared (_S) by several HCWs and used exclusively by night: the first one (named PEMD_S) was used in the PEMD by the whole team of on-call paediatricians excluding those working in the PICU, and the second one (named PICU_S) was used exclusively by paediatricians of the PICU. During the study, MPs were cleaned as usual (i.e., to the frequency and method usually used by the paediatrician without specific recommendation before or during the study) using disinfectant wipes that contain didecyldimethyl ammonium chloride as a biocide agent (WIP ANIOS EXCEL, Anios).

### 2.2. Sampling of MPs

One trained investigator (AC) performed the totality of the samplings. The MPs were rubbed with a nylon flocked swab allowing bacterial culture and viral molecular detection (e-swab ref 480CE, Copan, Brescia, Italy) using a standardised, reproducible prewritten procedure as described [11]: briefly, the e-swabs were dipped in the transport medium, all the surfaces (back, front and sides) and the buttons of the MPs were wiped the e-swabs (one per MP) and the swabs were placed in transport medium. The sampling of the MPs was performed every Tuesday morning, around 10:00 a.m., i.e., just after the medical staff when the paediatrician has already handled his/her MP, and before its cleaning.

### 2.3. Microbiological Analyses

We focused on the identification the most frequent bacteria described to be responsible for healthcare-associated infections in French paediatric settings, including *Staphylococcus aureus*, *S. capitis*, *Enterobacteriaceae* and *Pseudomonas* spp. [23,24,25,26]. A volume of 50 µL of the e-swab broth was plated onto an R2A agar plate (Oxoid, Life technologies, Courtaboeuf, France) and incubated at 30 °C for 5 days for quantifying total environmental and human flora. In addition, 20 µL of the broth were plated and incubated for 48 h onto the following selective plates: BBL CHROMagar Staph aureus (Becton Dickinson, le Pont de Claix, France), Chapman (bioMérieux, Craponne, France) and Uti Brillance (Oxoid, Life technologies, Courtaboeuf, France). Bacterial identification was performed using Microflex LT mass spectrometer (Bruker Daltonics, Champs sur Marne, France) on presumptive colonies of interest recovered from chromogenic media, on mannitol-positive colonies from Chapman medium (suggesting *S. aureus* or *S. capitis*) and on colonies exhibiting a viscous or mucoid phenotype on R2A plates. Antibiotic resistance patterns of strains were determined using the Vitek2 system (bioMérieux, Craponne, France). Determination of minimum inhibitory concentrations and detection of heteroresistance to vancomycin of *S. capitis* isolates were performed by using gradient antibiotic strips (E-tests, bioMérieux, Craponne, France) on Mueller Hinton (bioMérieux, Craponne, France) and BHI (Oxoid, Life technologies, Courtaboeuf, France) on Mueller Hinton and BHI agar, as recommended [27]. Strains of *S. capitis* were compared using arbitrarily primed PCR (AP-PCR) with primers 1 and Eric2 as previously described [28].

The virological analysis was performed by molecular techniques at the end of the study on frozen specimens. The extraction step was performed as described [11]. The genome of respiratory viruses [respiratory syncytial virus (RSV) A et B, influenza A et B, adenovirus [ADV], metapneumovirus, coronavirus 229E, NL63 and OC43, parainfluenza virus 1, 2, 3 and 4, bocavirus, enterovirus and rhinovirus] and gastro-intestinal viruses [norovirus GI and GII, rotavirus A, ADV F (serotype 40/41), astrovirus and sapovirus] were detected by RT-qPCR with Anyplex™II RV16 Detection kit and Allplex™ Gastrointestinal Full Panel Assay (Seegene, Eurobio, Courtaboeuf, France), respectively, according to the manufacturer’s instruction. Both kits are CE-marked, and their analytical performances were previously evaluated on clinical specimens [29,30]. For enteric viruses, the cycle threshold (Ct) values of amplification curves were available; for respiratory viruses, the targets were identified after analysis of the melting curves. The internal control of each kit was added at the extraction step to monitor the whole process as well as the absence of PCR inhibitor.

These investigations were all performed blind to the identity of the practitioner, and to the time of the sampling for viral genomes detection. All the results of the study were communicated to the practitioners only after the end of the study.

### 2.4. Hygiene Habits and Behavior

A questionnaire, similar to that previously used in a previous study from our team [11], was filled-in by all HCWs participating to the study before the beginning, immediately after the end of the study and four years later. This questionnaire includes questions about the use of DECT during care, the hand hygiene before and after its use and the stop of care provision to answer a call on DECT. All participants agreed to keep their hygiene habits unchanged during the inclusion period.

### 2.5. Statistics

Microsoft^®^ Excel 2016 was used for descriptive data, graphs, and tables. T-test and chi-square were performed for comparison groups on hygiene habits when appropriate (MedCalc^®^ v19.0.3). *p* value < 0.05 was considered significant.

### 2.6. Ethics

This study was approved by the Ethics Committee of the University Hospital of Saint-Etienne (IRB32N322016/CHUSTE).

## 3. Results

### 3.1. Detection of Viral Genomes and Bacterial Strains on MPs

A sum of 230 specimens (sampled once a week for 23 weeks on 10 MPs) was analysed during the study period; 145 samples (63.0%) were found contaminated by at least one pathogen. Multiple contaminations were observed in 49.0 % of the specimens (49 samples exhibited two agents, 13 three agents, 7 four agents and 2 five agents). A total of 247 pathogens were detected along the study, including 59 bacterial strains and the genome of 154 respiratory viruses and 34 enteric viruses (Table 1).

**Figure 1:**
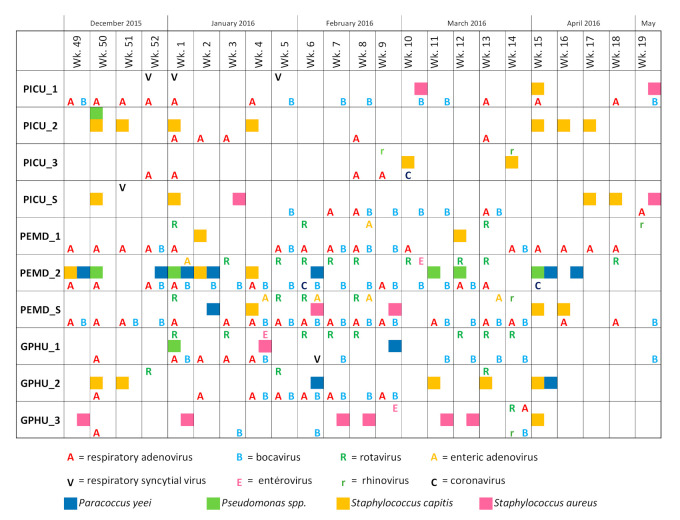
Overview of the contamination of the mobile phones (MPs) used by the paediatricians working in University Hospital of Saint-Etienne for 23 weeks. The names of the MPs are explained in the legend of Table 1. wk: week.

The kinetics of contamination of MPs through time is depicted in Figure 1 with the details of the recovered agents at each week for each MP. The MPs used in the PICU and GPHU were significantly less contaminated (46/92 positive samples, 50.0%, for PICU and 39/69, 56.5%, for GPHU) than those used in the PEMD (60/69, 87.0%) (*p* < 0.001 by chi2 test). On the sample of phones used in PEMD, multiple contaminations were frequent (37/69, 61.7%).

### 3.2. Contamination with Bacteria

Common flora was found on 97% of MPs, with 92.3% of them harbouring less than 10^3^ CFU/mL and 7.7% showing bacterial loads ranging from 10^3^ to 1.4 × 10^4^ CFU/mL of e-swab broth. *Staphylococcus* spp. was the most predominant bacterial genus; *S. capitis* was detected in 28 cases and *S. aureus* in 13 cases. No resistance to methicillin was found for all *S. aureus* strains. *S. capitis* strains were all sensitive to vancomycin with minimum inhibitory concentrations ≤ 1 mg/L and no heteroresistance was detected using E-tests (data not shown). AP-PCR using 2 different primers allowed us to discriminate between strains of *S. capitis* isolated from the different MPs and indicated that these strains were different from the NRCS-A resistant clone (data not shown). *S capitis* was isolated on every DECT during the study period while *S. aureus* was identified mainly on one phone (GPHU_3 in 5 samples). Gram-negative bacteria were dominated by *Pseudomonas* spp. (13 cases).

### 3.3. Detection of Viral Genomes

Overall, the viral loads on MPs were low, with Ct values comprising between 33.4 and 39.5 for rotavirus and between 37.6 and 40.0 for enteric ADV and noted between + and ++ for positive with respiratory viruses. The most frequent genomes that were detected were those of respiratory ADVs (*n* = 66), bocaviruses (*n* = 63) and rotaviruses (*n* = 28). The genome of enteric ADVs, RSVs, rhinoviruses, coronaviruses and enteroviruses were detected in 6, 5, 5, 3 and 3 cases, respectively. Respiratory ADV and bocavirus genomes were co-detected on the same sample in 32 of 97 cases (33%) for which at least one of them was present. Rotavirus RNA was also frequently detected in association with other viral genome(s) (28/34, 82.4%). Enteric ADV genomes were all detected in combination with respiratory ADV and bocavirus ones, and they were only detected on MPs from PEMD. RSV RNA was mostly detected on MPs from the PICU, one of them (PICU_1) having been found positive 3 times during the study period; in contrast, enteric viruses were not detected in these devices. Co-detection of viral genomes was frequently observed, especially in the GPHU (*n* = 14) and the PEMD (*n* = 31). Co-detection of viral genomes and bacteria was also common in the PEMD (14 cases).

The wave of MPs contamination could be perfectly superimposed onto the seasonal epidemic (Figure S1). It was particularly true for rotaviruses that were detected on MPs from week 1 to week 14, and for enteric adenoviruses that were detected on MPs along all the period studied.

### 3.4. Transfer of Contamination

**Figure 2:**
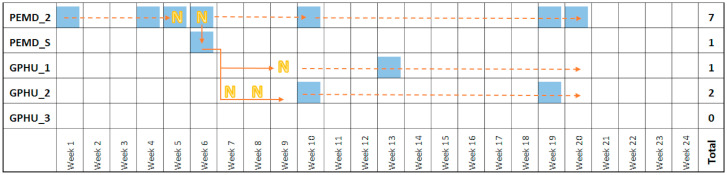
Transfer of contamination: the case of *Paracoccus yeei*. The names of the MPs are explained in the legend of Table 1. N: phone used the night by the paediatrician on duty.

A strain of *Paracoccus yeeii* was isolated from PEMD_2; it was initially identified because of its mucoid and viscous phenotype on R2A medium mimicking a Gram-negative bacterium drawing our attention. The follow-up of the presence of this species, further identified by mass spectrometry analysis, on the 10 DECTs during the study period showed its transfer from PEMD_2 to PEMD_S, the phone shared by night by the paediatricians on duty at the PEMD (Figure 2). The paediatricians that handled GPHU_1 and GPHU_2 also used the PEMD_S phone when they worked by night in the PEMD *P. yeeii* persisted on GPHU_1 and GPHU_2 MPs up to one month later (Figure 2). By contrast, this bacterium was not present on the MPs used in the PICU, the paediatricians of this ward not sharing their MP with colleagues of the other wards. Despite its absence of pathogenicity, this strain illustrates the ability of infectious agents to be transmitted via hand carriage and inert material (and vice versa) from a ward to another.

### 3.5. Hygiene Behaviours

At the beginning of the study, half of the 8 paediatricians declared that they clean their MP at least once a week, whereas the other 4 practitioners declared that they never clean their MP. After communication of the results of MPs contamination, all the participants declared cleaning their MP at least once a week, with up to five times a week for the most active ones. The mean number of performing MP cleaning increased by 3.3-fold between the beginning and the end of the study (Table 2), and this effect persists 4 years later. Table 2 also shows that this study modified significantly other aspects of the relationship between phoning and care, including increased hand hygiene after MP use and limitation of MP use during clinical examination.

## 4. Discussion

This longitudinal study reports for the first time the sequential contamination of MPs used by paediatricians within their usual care setting including an entire winter season. As already published [3,8,11,12,19], our study highlights the huge contamination rate of paediatricians’ MPs and, as illustrated in Figure 1, the contamination persists through time. The MPs used in the PEMD, which is considered a gateway of epidemic pathogens [31], were shown to be significantly the most contaminated ones; the use of this object, even during and after care [11], leads to a frequent transfer of pathogens on its surface [32]. The MPs used in the PICU were found to be less contaminated by viruses, with notably no enteric genomes (Table 1). However, they were not free of contamination, suggesting that efforts can still be made in terms of hygiene, even in this confined service.

As MPs are not sterile devices and are frequently touched by hands, all those tested in this study were shown to harbour saprophytic skin flora, especially coagulase negative staphylococci, whereas Gram-negative bacteria were far less frequently detected, as already described [33]. Our investigations focused on some bacteria that were shown to be responsible for severe healthcare-associated infections in French paediatric wards, including *S. aureus*, Gram-negative isolates [23] or more recently, *S. capitis* [26]. This choice explains the restricted number of bacterial species reported in our study in comparison to others [5,32]. No strain of methicillin-resistant *S. aureus* was found on MPs and during the study period no MRSA infection was reported. Attention was paid to *S. capitis* because a clonal strain, with reduced susceptibility to vancomycin, named NRCS-A, was described as an emerging cause worldwide of late-onset sepsis in preterm neonates [26]; this clone had been identified earlier in a few cases of infections in our hospital. In the present study, all the strains detected on MPs were found to be sensitive to vancomycin. However, the high prevalence of the *S. capitis* species on MPs or hands, not previously reported, could constitute a reservoir participating to the dissemination of strains of *S. capitis* with reduced susceptibility to vancomycin in wards where the NRCS-A clone is endemic.

The transmission of bacteria between MPs over time was clearly documented by a strain of *P. yeei* that was detected incidentally on several MPs as a marker of cross-contamination. This bacterium, usually found in soil, is mainly considered as non-pathogenic for humans although some opportunistic infections have been described [34]. Detected during the first week of the study on PEMD_2, it was observed on different MPs during the survey (Figure 2), the transmission between MPs having probably occurred during the sharing of phones by on-call paediatricians.

The virological analyses detected mainly the genome of naked viruses, which was predictable due to their ability to persist in the environment [14]. The period with the higher contamination by viral genomes was comprised between January to April, matching the peak of winter epidemics [21,22]. Respiratory viruses were the most frequently detected ones, mainly bocavirus and ADV. Enveloped viruses, like influenza viruses and coronaviruses were rarely detected, although some of them were suspected to spread via innate surfaces [20], suggesting that MPs may not be the major route of transmission. To our opinion, this information is also to be highlighted in the context of current pandemic of SARS-CoV-2; further studies must be performed to assess this hypothesis.

Concerning enteric viruses, the main genomes detected were those of rotaviruses and ADVs, which is in accordance with their persistence on hospital surfaces [13,14,19]. As also reported in our previous study [11], we detected no norovirus genome, which may be explained by a lack of sensitivity of the molecular kit that was used [30].

## 5. Conclusions

In contrast to the forest of publications that are snapshots of a one-day sampling, this longitudinal study highlights the persistence of viral genomes together with bacteria as well as the ability of some of them to be transferred from one MP to another, probably by hands. This study allowed the medical staff to become more aware of the contamination risk of these devices, leading to a dramatic effect on the hygiene behaviour of the clinicians. In the future, the role of MPs as vectors of nosocomial infection needs to be better investigated. Decontamination procedures of these devices must be promoted.

## Figures and Tables

**Table 1:** Viruses and bacteria detected on voluntary paediatrician’s mobile phones (MPs) for 23 weeks in the University Hospital of Saint-Etienne. Number of positive samples among the total of 230 ones performed along the 23 weeks of the study. The MPs were named according to the department they were used: paediatric intensive care unit (PICU), paediatric emergency department (PEMD) or general paediatric hospitalization unit (GPHU). MPs numbered 1 to 8 were nominative ones; PICU_S and PEMD_S were shared by all on-call paediatricians.

	PICU_1	PICU_2	PICU_3	PICU_S	PEMD_1	PEMD_2	PEMD_S	GPHU_1	GPHU_2	GPHU_3	Total
Respiratory viruses *											
Adenovirus	9	5	4	4	15	8	16	5	7	2	75
Bocavirus	7			6	6	13	14	8	6	3	63
Respiratory syncytial virus	3			1				1			5
Rhinovirus			2		1		1			1	5
Enterovirus						1		1		1	3
Coronavirus			1			2					3
Total	19	5	7	11	22	24	31	15	13	7	154
Enteric viruses *											
Rotavirus					3	9	4	8	3	1	28
Adenovirus					1	1	4				6
Total					4	10	8	8	3	1	34
Bacteria											
*Staphylococcus aureus*	2			2			2	1		6	13
*Staphylococcus capitis*	1	7	2	4	2	3	3		5	1	28
*Pseudomonas aeruginosa*		1				5		1			7
*Paracoccus yeei*						7	1	1	2		11
Total	3	8	2	6	2	15	6	3	7	7	59
Total	**22**	**13**	**9**	**17**	**28**	**49**	**45**	**26**	**23**	**15**	**247**

* Influenza viruses A and B, parainfluenza virus, metapneumovirus, norovirus, astrovirus, and sapovirus genome were not detected on the analysed MPs.

**Table 2:** Hygiene behaviours of the 8 healthcare workers (HCWs) who participated to the study.

	Before the Study (*n* = 8)	After the Study (*n* = 6)	*p* value Comparing the Percentage before/after the Study	4 Years after the End of the Study (*n* = 6)
HCWs who wash their hands before using the MP (%) *	11.1	16.6	0.68	11.2
HCWs who wash their hands after using the MP (%) *	16.6	43.7	**0.03**	75
HCWs who stop clinical examination to answer a phone call (%) *	72.2	41.2	**0.04**	33
HCWs who wash their hands before pursuing the examination (%) *	42.5	68.7	0.07	75
Number of calls received every day (mean (SD)) †	12 (3.8)	12.5 (4.1)	0.97	11.6 (4.0)
Number of times the MP is cleaned every month (mean (SD)) †	2.3 (4.1)	7.6 (6.3)	**0.02**	8.1 (10.6)

* Chi-square test. † T test assuming the normal hypothesis. MP: mobile phone; SD: standard deviation. Statistically significant *p* values are shown in bold.

## Supplementary Materials

Supplementary materials can be found at https://www.mdpi.com/2076-2607/8/12/2011/s1.
