# Supplementary material for: Longitudinal Study of Viral and Bacterial Contamination of Hospital Pediatricians’ Mobile Phones
Source: Microorganisms. 2020 Dec 16;8(12):2011. doi: 10.3390/microorganisms8122011 (PMC7766489; doi:10.3390/microorganisms8122011)
Supplement: Supplementary file 1 [file microorganisms-08-02011-s001.pdf]

Supplementary Materials

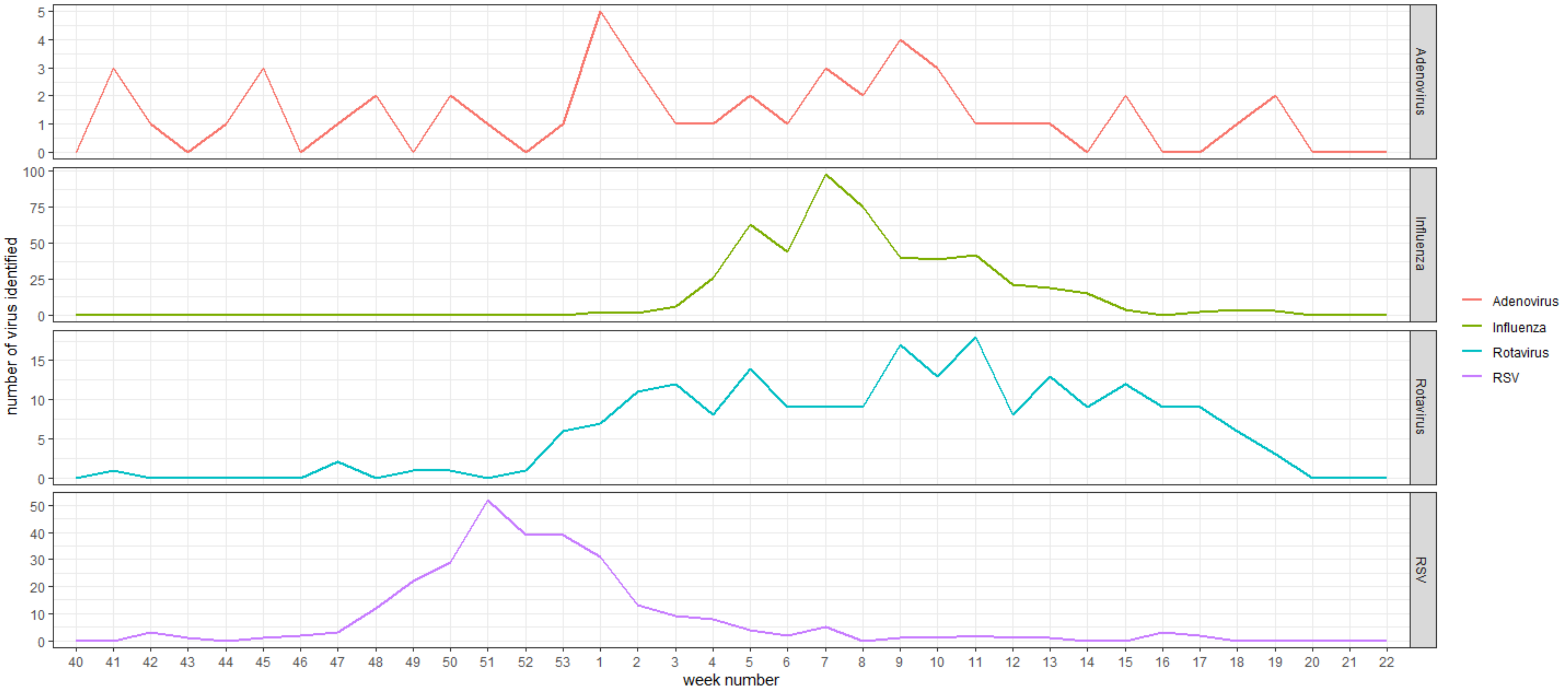

Figure S1. Epidemiological data in our centre during the study period.

© 2020 by the author. Licensee MDPI, Basel, Switzerland. This article is an open access article distributed under the terms and conditions of the Creative Commons Attribution (CC BY) license (http://creativecommons.org/licenses/by/4.0/).
